# Economic evidence on provider-level implications of direct posterior amalgam alternatives following the EU phase-out: a scoping review

**DOI:** 10.1136/bmjopen-2026-118949

**Published:** 2026-05-19

**Authors:** Johannes Oesterreicher, Marco M Herz

**Affiliations:** 1 Department for Conservative Dentistry, University of Tübingen, Tübingen, Germany

**Keywords:** Dentistry, HEALTH ECONOMICS, Health Care Costs, Health policy

## Abstract

**Abstract:**

**Objective:**

To map and characterise economic evaluations (EEs) and provider-level cost/resource reporting related to direct posterior restorative alternatives following the European Union (EU) dental amalgam phase-out.

**Design:**

Scoping review in accordance with Preferred Reporting Items for Systematic Reviews and Meta-Analyses extension for Scoping Reviews (PRISMA-ScR) guidance.

**Data sources:**

PubMed, Embase, Web of Science, CINAHL and LIVIVO (ZB MED search portal for life sciences (Germany)).

**Eligibility criteria:**

Peer-reviewed studies (English/German; 1 January 2021–22 February 2026) reporting EEs or cost/resource-use components for direct posterior restorations in permanent teeth.

**Data extraction and synthesis:**

Data were charted using a predefined extraction framework focusing on analytic perspective, time horizon, modelling approach and granularity of provider-level resource components. Two reviewers independently screened records and charted data; disagreements were resolved by discussion and, if needed, by consultation with a third reviewer.

**Results:**

Six studies met the inclusion criteria (four model-based or trial-based EEs; two cross-sectional surveys). Most analyses adopted payer, societal or mixed perspectives and used decision-analytic models with medium- to long-term horizons. Outcomes included cost per complication-free month, cost per tooth-year retained and lifetime cost projections. Detailed provider-level reporting (eg, chair time, personnel allocation, overheads or warranty-related retreatment burden) was limited, and survey evidence relied on self-reported estimates. Heterogeneity in methods and metrics precluded quantitative synthesis.

**Conclusions:**

Economic evidence regarding direct posterior restorative alternatives after the EU amalgam phase-out is sparse and primarily based on modelled or reimbursement-derived inputs from payer, societal or mixed perspectives, rather than explicitly measured provider-level microcosting. Greater transparency in analytic perspective and microcosting components may support evidence-informed adaptation to restorative material substitution policies.

**STRENGTHS AND LIMITATIONS OF THIS STUDY**

- This scoping review was conducted and reported in accordance with Preferred Reporting Items for Systematic Reviews and Meta-Analyses extension for Scoping Reviews (PRISMA-ScR) guidance.
- A comprehensive search across five databases was performed, and screening and data charting were conducted independently by two reviewers.
- The review maps not only economic evaluation types and perspectives but also the granularity of provider-level resource reporting.
- Protocol registration was retrospective.
- The small number of eligible studies, methodological heterogeneity and exclusion of grey literature may limit the completeness and comparability of the evidence base.

## Introduction

 The phase-out of dental amalgam within the European Union (EU) represents a major regulatory shift in restorative dental care. As of 1 January 2025, the use of dental amalgam has been largely prohibited under amendments to the EU Mercury Regulation, reflecting environmental and public health objectives aligned with the Minamata Convention on Mercury.^1^,^3^ This development requires healthcare systems to ensure the continued provision of durable and economically sustainable restorative care using mercury-free alternatives. Across EU member states, restorative dental care is embedded within diverse reimbursement systems and regulatory frameworks. While specific requirements differ, many publicly funded systems impose performance expectations on restorative materials. For example, in Germany, § 136a of the Sozialgesetzbuch Book V (German Social Code Book V) stipulates a 2-year warranty period for dental fillings provided under statutory health insurance (Gesetzliche Krankenversicherung (German Statutory Health Insurance)).^4^ Similar regulatory and reimbursement constraints across EU countries underscore the need for durable and economically sustainable amalgam alternatives.

Restorative materials replacing amalgam in posterior load-bearing regions must therefore reliably perform for at least 2 years under routine clinical conditions. This statutory requirement elevates short- and medium-term clinical effectiveness from a technical outcome to a health policy-relevant benchmark.

Resin-based composite restorations have become the principal alternative to amalgam in posterior teeth. Systematic reviews and meta-analyses report favourable survival rates for Class I and II composite restorations, with annual failure rates commonly ranging between 1% and 3%, depending on patient risk profiles and cavity characteristics.^5 6^ Long-term practice-based data support the clinical viability of posterior composites, including multisurface restorations, when adhesive protocols are appropriately applied.^7 8^ A comprehensive meta-analysis confirmed overall durability while highlighting heterogeneity in study design and reporting.^5 9^

Comparative evidence suggests that although amalgam historically demonstrated slightly longer median survival in some datasets, differences between amalgam and contemporary resin-based materials are context-dependent and supported by low- to moderate-certainty evidence.^10 11^ Importantly, reported survival outcomes for modern composite systems generally exceed the 2-year minimum warranty requirement under German statutory conditions.^6 10^

Beyond clinical survival, the amalgam phase-out has implications for dental practice organisation and resource allocation. Adhesive composite placement typically involves additional procedural steps—such as isolation, incremental placement and light curing—which may increase chairside time compared with amalgam restorations.^12 13^ Provider-level costs, therefore, extend beyond material expenses to include personnel time, equipment use and potential reintervention rates. However, comprehensive economic evaluations (EEs) in restorative dentistry remain only partially captured, and cost reporting is often secondary to clinical endpoints.^14^,^16^ Previous critical reviews have highlighted heterogeneity and incomplete cost reporting in restorative research.^11 14^

Despite the regulatory elimination of dental amalgam and statutory durability requirements in publicly funded systems, the extent to which provider-level costs and resource implications of direct posterior restorative alternatives are systematically reported remains unclear. Existing literature predominantly focuses on survival outcomes, while economic components relevant to routine clinical practice are inconsistently described. In this review, we distinguish between the provider-level perspective and the provider-level costing. The provider perspective refers to the analytic viewpoint, that is, which stakeholder bears the costs. Provider-level costing, in contrast, refers to how costs are measured, including the level of detail in reporting resource use such as chair time, personnel input and overhead allocation.

The aim of this scoping review was to systematically map and characterise the available economic evidence on direct posterior restorative alternatives to dental amalgam, with particular focus on how provider-level resource components are reported. Specifically, the review sought to identify (1) the types of EEs conducted, (2) the analytic perspectives adopted and (3) the extent to which granular provider-level cost components relevant to regulatory implementation are described.

## Materials and methods

### Study design

This review was conducted as a scoping review to map and characterise the available economic evidence on direct posterior restorative alternatives to dental amalgam in the context of the EU phase-out. A scoping approach was chosen because the evidence base was expected to be heterogeneous with respect to study designs, analytic perspectives, costing approaches, time horizons and outcome metrics, and because comprehensive provider-level cost reporting was anticipated to be sparse.^17 18^ The review was undertaken and reported in accordance with the Preferred Reporting Items for Systematic Reviews and Meta-Analyses extension for Scoping Reviews (PRISMA-ScR). The objectives were to identify and categorise (1) the types of EEs, and cost and resource reporting approaches used, (2) the analytic perspectives adopted (provider, payer and societal) and (3) the extent to which granular provider-level resource components—such as chair time, personnel inputs, overheads and short-term reintervention burden relevant to warranty periods—were described. Consistent with scoping review methodology, the aim was descriptive mapping rather than critical appraisal of study quality or quantitative synthesis of economic outcomes.

### Eligibility criteria

Eligibility criteria were defined a priori using the Population–Concept–Context (PCC) framework recommended for scoping reviews.

*Population*: studies involving permanent posterior teeth (premolars and molars) restored with direct restorative materials were eligible. Studies exclusively focusing on primary dentition or non-load-bearing anterior restorations were excluded.

*Concept*: the review focused on provider-level economic aspects, including reported material costs, treatment time, resource utilisation, reintervention rates and formal or partial EEs. Studies were eligible if they reported any cost-related component relevant to routine dental practice, regardless of whether a full EE was conducted. Laboratory-only investigations without clinical or cost implications were excluded.

*Context*: the context of interest was routine clinical dental care involving direct restorative materials used as alternatives to dental amalgam. Studies addressing indirect restorations (eg, inlays, onlays and crowns) were excluded unless direct restorative materials were explicitly evaluated. No geographical restrictions were applied; however, regulatory relevance to publicly funded health systems was considered during data synthesis.

Additional inclusion criteria comprised peer-reviewed publications in English or German published between 1 January 2021 and 22 February 2026. Conference abstracts, editorials, commentaries and non-peer-reviewed reports were excluded.

### Information sources

A comprehensive literature search was conducted in the following electronic databases: PubMed, Embase, Web of Science Core Collection, CINAHL and LIVIVO (ZB MED search portal for life sciences (Germany)). These databases were selected to capture biomedical, clinical, health services and German-language literature relevant to restorative dentistry and EE. The search covered publications from 1 January 2021 to 22 February 2026. No geographical restrictions were applied. In addition to electronic database searches, reference lists of included studies were screened to identify potentially relevant additional publications. Given the focus on peer-reviewed EEs and reporting standards, grey literature was not systematically searched; this may have resulted in omission of policy documents, reimbursement reports or unpublished economic analyses relevant to implementation.

### Search strategy

The search strategy was developed to identify studies addressing provider-level cost and resource implications of direct posterior restorative materials used as alternatives to dental amalgam. Controlled vocabulary terms and free-text keywords were combined using Boolean operators. Search terms included concepts related to direct restorative materials (eg, composite resins, bulk-fill materials, glass ionomer cements (GICs)), posterior restorations (eg, molars, Class II) and economic or resource-related outcomes (eg, costs, cost-effectiveness, resource utilisation and treatment time).

The search strategy was initially developed for PubMed and subsequently adapted to the syntax and indexing terms of the other databases. Filters were applied to restrict results to the predefined publication period from 1 January 2021 to 22 February 2026 and to publications in English or German where applicable.

The full search strategies for all databases are provided in online supplemental table S2. We limited inclusion to publications from 2021 onwards to capture economic analyses reflecting contemporary restorative materials, clinical workflows and reimbursement contexts in the period of regulatory transition towards the EU dental amalgam phase-out.

### Selection of sources of evidence

All records identified through the database searches were exported into a reference management software program, and duplicates were removed prior to screening. The selection process was conducted in two stages. In the first stage, titles and abstracts were independently screened by two reviewers against the predefined eligibility criteria. In the second stage, full texts of potentially relevant articles were retrieved and assessed independently by the same reviewers for final inclusion.

Disagreements at any stage of the screening process were resolved through discussion. If consensus could not be reached, a third reviewer was consulted. Reasons for exclusion at the full-text stage were documented. The study selection process is presented in a Preferred Reporting Items for Systematic Reviews and Meta-Analyses (PRISMA) flow diagram.

### Data charting process

Data charting was performed using a standardised data extraction form developed specifically for this review. The form was designed to capture study characteristics and cost-related variables relevant to provider-level resource use and reporting granularity. Prior to full data extraction, the form was pilot tested on a small sample of included studies and refined to ensure clarity and consistency. Two reviewers independently extracted data from the included studies. The extracted data were compared, and discrepancies were resolved through discussion. If necessary, consensus was achieved through consultation with a third reviewer. Consistent with scoping review methodology, the data charting process aimed to map and categorise the available evidence rather than to critically appraise study quality or synthesise effect estimates.

### Data items

The following data items were extracted from each included study:

- Bibliographic information (author, year of publication and country of study).
- Study design and methodological approach.
- Type of restorative material evaluated.
- Comparator material, where applicable.
- Clinical context (eg, Class I or Class II restorations, posterior teeth).
- Economic perspective reported (eg, provider, payer and societal).
- Type of EE (eg, full EE, partial cost analysis and cost description only).
- Cost components included (eg, material costs, personnel costs, treatment time and overhead costs).
- Resource utilisation measures (eg, chair time, number of visits).
- Reintervention or replacement rates, if reported.
- Time horizon of the analysis.
- Main economic findings relevant to routine dental practice.

Where economic data were incompletely reported, this was documented descriptively.

### Synthesis of results

Given the anticipated heterogeneity in study designs, economic reporting formats and outcome measures, no quantitative meta-analysis was performed.

Included studies were grouped according to study design and type of economic reporting, distinguishing between full EEs, partial cost analyses and studies reporting isolated cost or resource components. Cost components and perspectives were categorised to identify patterns in reporting practices and methodological approaches.

Findings were summarised narratively and presented in structured tables to provide an overview of reported cost elements, treatment time measures and reintervention data relevant to routine dental practice. Where appropriate, differences in reporting by geographic region or health system context were described.

The synthesis focused on identifying evidence gaps, methodological heterogeneity and implications for future economic research in restorative dentistry.

### Reporting framework and protocol

This scoping review was conducted and reported in accordance with PRISMA-ScR. The PRISMA-ScR checklist is provided in online supplemental table S1. Eligibility criteria, the search strategy and the data charting framework were agreed within the team before screening started. The protocol was registered retrospectively on the Open Science Framework (OSF; https://osf.io/938yq) after completion of the search and data charting but prior to manuscript submission, in order to document methodological decisions transparently. Any deviations from the planned approach are described in the manuscript where applicable.

### Critical appraisal of individual sources of evidence (if applicable)

No formal critical appraisal or risk-of-bias assessment was undertaken, consistent with scoping review methodology.^19 20^ Instead, key methodological limitations and sources of uncertainty were summarised descriptively to support the interpretation of findings.

### Patient and public involvement

Patients or the public were not involved in the design, conduct, reporting or dissemination plans of our research.

## Results

### Study selection

The database search identified a total of 413 records, of which 279 remained after removal of duplicates. Following title and abstract screening, 245 articles were excluded.

A total of 34 full-text articles were assessed for eligibility. Of these, 28 studies were excluded at the full-text stage for predefined reasons (online supplemental table S3). The most common reason for exclusion was the absence of economic outcomes. Other reasons included in vitro design, wrong population, non-clinical study design and non-primary research. A detailed list of excluded studies with reasons is provided in online supplemental table S4.

**Figure 1:**
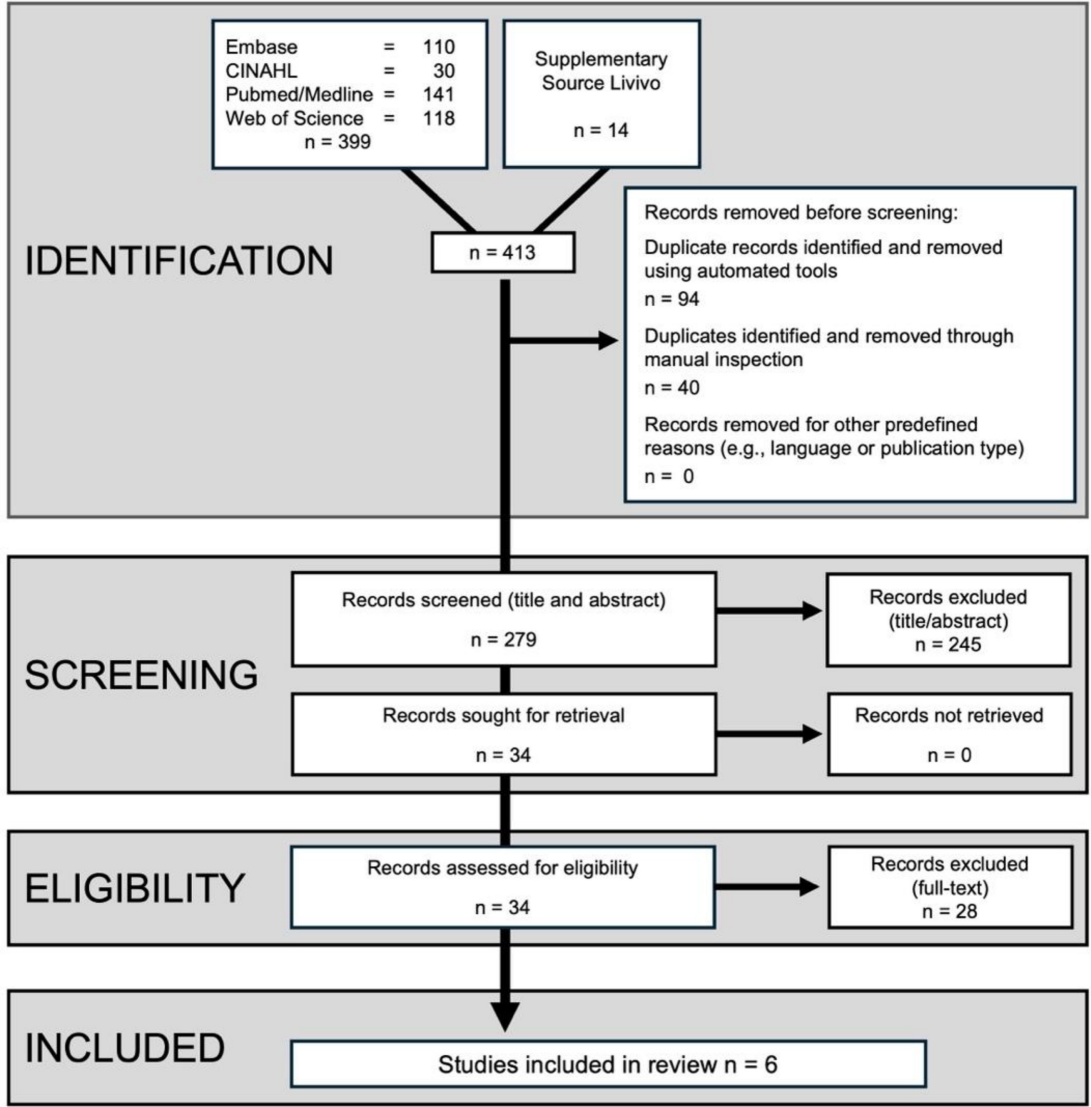
Preferred Reporting Items for Systematic Reviews and Meta-Analyses (PRISMA) flow diagram.

Ultimately, six studies met the inclusion criteria and were included in the qualitative synthesis.^1516 21^,^24^ The study selection process is summarised in figure 1.

### Study characteristics

Of the six included studies, four were formal EEs, and two were survey-based studies examining provider-level economic implications (table 1).

**Table 1:** EE of amalgam alternatives—summary of included studies

Study	Country	Design and model	Comparator	Perspective and time horizon	Economic outcome	EE	Main economic finding
Schwendicke *et al* (trial-based)^16^	Four countries	RCT+trial-based CEA (3 years)	GH restoration versus composite	Payer; 3 years	Cost per complication-free month	CEA	GH less costly; similar effectiveness
Schwendicke *et al* (model)^15^	Germany	Lifetime Markov model	GH restoration versus composite	Mixed payer; lifetime	ICER per tooth-year retained	CEA	Composite slightly more effective but more costly; GH likely cost-saving
Han *et al*^23^	South Korea	Markov microsimulation	RC versus GIC	Patient/system/societal; 15 years	ICER per tooth-year	CEA	RC cost-effective; dominant with insurance expansion
Bailey *et al*^21^	England (NHS)	Lifetime microsimulation	Amalgam versus composite	NHS+societal; lifetime	Lifetime cost and survival	CEA	Amalgam dominant (lower cost, longer survival)
Bailey *et al*^22^	UK	Survey	Amalgam versus composite	Provider-level; cross-sectional	Treatment time and fees	No EE	Composite ∼45% more time and cost
Usenik *et al*^24^	Slovenia	Survey	Amalgam versus alternatives	Provider-level/policy	Reimbursement impact	No EE	Coverage strongly influences material choice

CEA, cost-effectiveness analysis; EE, economic evaluation; GH, glass hybrid; GIC, glass ionomer cement; ICER, incremental cost-effectiveness ratio; NHS, National Health Service; RC, resin composite; RCT, randomised controlled trial.

Three studies employed decision-analytic modelling approaches with lifetime horizons, while one study conducted a trial-based cost-effectiveness analysis (CEA) alongside a randomised clinical trial with a 3-year follow-up. The studies were conducted in multinational university settings, Germany, South Korea, England within the National Health Service (NHS), the UK primary care setting and Slovenia.

Comparators varied across studies and included:

- Glass hybrid (GH) versus resin composite (RC).^15 16^
- RC versus GIC.^23^
- Amalgam versus composite.^20 21^
- Amalgam versus alternative restorative materials.^24^

Time horizons ranged from 3 years to lifetime modelling approaches.

### Economic outcomes

#### GH versus RC

The multinational randomised clinical trial with trial-based CEA demonstrated that GH restorations were generally less costly than RC restorations over a 3-year period, while differences in effectiveness were minimal. In the lifetime Markov model based on German healthcare costs, RC showed slightly longer tooth retention but at higher lifetime costs. The incremental cost-effectiveness ratio per additional tooth-year retained was modest. Results were reported as sensitive to parameter uncertainty; however, the type of sensitivity analysis (probabilistic versus deterministic) was not consistently specified across studies. In several scenarios, the GH was cost-saving or associated with negligible effectiveness loss. Across both studies, GH restorations were reported to have lower costs, while the effectiveness differences compared with RC were small (table 2).

**Table 2:** Cost-effectiveness results of included economic evaluations across all comparator groups

Study	Country/setting	Comparator	Model/design	Perspective	Time horizon	Economic outcome	Main economic conclusion
Schwendicke *et al*^16^	Multinational (four university clinics)	GH versus RC	RCT+trial-based CEA	Payer	3 years	Cost per complication-free month	GH less costly with comparable effectiveness
Schwendicke *et al*^15^	Germany	GH versus RC	Lifetime Markov model	Mixed payer	Lifetime	ICER per tooth-year retained	Composite slightly more effective but more costly; GH likely cost saving
Han *et al*^23^	South Korea	RC versus glass ionomer cement	Markov microsimulation	Patient/system/limited societal	15 years	ICER per tooth-year	RC cost-effective; dominant under expanded insurance coverage

CEA, cost-effectiveness analysis; GH, glass hybrid; ICER, incremental cost-effectiveness ratio; RC, resin composite; RCT, randomised controlled trial.

#### RC versus GIC

A microsimulation study conducted in South Korea evaluated RC versus GIC in cancer patients requiring restorative care.^23^

RC increased tooth retention compared with GIC. From a limited societal perspective, RC was cost-effective and became cost-saving (dominant) under expanded insurance coverage scenarios.^23^ Without insurance expansion, RC incurred higher costs but remained within acceptable cost-effectiveness thresholds. The cost-effectiveness results varied depending on insurance coverage scenarios.

#### Amalgam versus composite restorations

A lifetime microsimulation study conducted within the English NHS found that amalgam restorations dominated composite restorations.^21^ Amalgam was associated with lower lifetime costs, longer restoration and tooth survival, and fewer treatment visits. The model projected higher healthcare costs and shorter tooth survival for composite compared with amalgam under the analysed NHS conditions.

A UK primary care survey further reported that composite restorations required approximately 45% more clinical time and were associated with higher charges compared with amalgam.^22^

#### Granularity of provider-level economic reporting

Across included studies, the level of granularity in reporting provider-level resource components varied substantially. While four studies conducted formal EEs using decision-analytic modelling or trial-based approaches, explicit microcosting of chair time, personnel allocation, overhead structures or warranty-related retreatment costs was rarely described. Only one survey-based study reported self-reported treatment time differences between restorative materials.

Across studies, economic reporting was predominantly based on system-level modelling and reimbursement-based costing, with no study reporting explicit microcosting based on direct measurement of workflow inputs. Costing approaches differed markedly across studies. Model-based studies primarily relied on aggregated reimbursement data or assumed cost inputs, rather than direct measurement of resource use.

### Provider-level reimbursement and incentive factors

A cross-sectional survey conducted in Slovenia demonstrated that reimbursement structures and financial incentives significantly influenced restorative material choice.^24^ Lower compliance with amalgam phase-down policies was observed in financially constrained clinical settings.

Both surveys reported associations between reimbursement structures, treatment time and restorative material choice (table 3).

**Table 3:** Provider-level economic findings

Study	Country	Study type	Comparator	Economic focus	Key findings	Reported economic association
Bailey *et al*^22^	UK	Cross-sectional survey	Amalgam versus Composite	Treatment time and fees	Composite required ~45% more clinical time and higher charges	Increased resource use when replacing amalgam with composite
Usenik *et al*^24^	Slovenia	Cross-sectional survey	Amalgam versus alternatives	Reimbursement structures and compliance	Financial barriers influenced material choice and phase-down compliance	Associations between reimbursement structures and material choice were reported

### Methodological considerations and sources of uncertainty

Methodological characteristics and key sources of uncertainty are summarised in table 4. The identified evidence base was predominantly composed of model-based EEs, supplemented by one trial-based analysis and two survey-based studies. Model-based EEs relied on structural assumptions and long-term extrapolation of short-term clinical data. Sensitivity analyses were reported across studies; however, the type of approach (probabilistic or deterministic) was not always explicitly specified. In addition, reporting on model validation and uncertainty characterisation was limited, which may constrain full assessment of robustness. Trial-based economic analysis was limited by follow-up duration and setting-specific cost inputs. Survey-based studies provided descriptive insights into treatment time and reimbursement influences but were subject to self-report and sampling biases.

**Table 4:** Methodological characteristics and key sources of uncertainty of included studies

Study	Study design	Economic approach	Time horizon	Key assumptions/data sources	Main sources of uncertainty	Notes on transferability
Schwendicke *et al* (trial-based)^16^	Multinational split-mouth RCT+trial-based CEA	Within-trial CEA	3 years	Payer fee catalogues; PPP adjustments; complications as outcome	Limited follow-up duration; university clinic setting; interim data	Clinical setting may not reflect routine primary care
Schwendicke *et al* (model)^15^	Lifetime Markov model (Germany)	Model-based CEA	Lifetime	Transition probabilities derived from 3-year RCT; German cost inputs	Structural assumptions for long-term extrapolation; hazard assumptions; cost structure context-specific	Cost inputs specific to the German mixed-payer system
Han *et al*^23^	Markov microsimulation	Model-based CEA	15 years	Claims-based cohort data; meta-analytic effectiveness inputs	External validity of effect estimates; insurance scenario modelling; parameter uncertainty	Based on the South Korean insurance structure
Bailey *et al*^21^	Microsimulation model (NHS England)	Lifetime cost-effectiveness modelling	Lifetime	NHS pathway modelling; composite failure assumptions; national claims validation	Comparative effectiveness uncertainty; constant hazard assumptions in parts of the model	Highly specific to NHS reimbursement and care pathways
Bailey *et al*^22^	Cross-sectional survey	Descriptive cost/time reporting	Cross-sectional	Self-reported treatment time and fees	Response bias; recall bias; lack of objective cost verification	Reflects the UK primary care context
Usenik *et al*^24^	Cross-sectional survey	Descriptive policy/practice analysis	Cross-sectional	Self-reported reimbursement and material choice	Selection bias; social desirability bias; context-specific regulation	Based on the Slovenian reimbursement framework

CEA, cost-effectiveness analysis; NHS, National Health Service; PPP, purchasing power parity; RCT, randomised controlled trial.

Across studies, transferability of findings may be influenced by jurisdiction-specific reimbursement structures, insurance coverage and care pathways.

## Discussion

### Principal findings

This scoping review mapped the current economic evidence relating to provider-level implications of direct posterior restorative alternatives following the EU dental amalgam phase-out. Although no geographical restrictions were applied, only six eligible studies were identified, comprising four formal EEs and two survey-based analyses. This limited number and heterogeneity of studies highlight a substantial gap in the evidence base, indicating that provider-level economic implications remain underexplored internationally. Most EEs adopted payer, societal or mixed analytic perspectives and relied on decision-analytic modelling or trial-based cost-effectiveness analyses. Reported outcomes included cost per complication-free month, cost per tooth-year retained and lifetime cost projections. While consistent with established health economic frameworks,^25 26^ these approaches primarily inform system-level efficiency rather than operational implications for routine dental practice.

Comparators, time horizons and modelling assumptions varied substantially. Conclusions ranged from the dominance of amalgam under specific national conditions to the cost-effectiveness of composite or GH restorations under defined insurance scenarios. This variability underscores the context sensitivity of EE and the importance of clearly defined analytic perspectives.^27^

Most notably, detailed reporting of provider-level resource components remained limited. Even where modelling approaches were methodologically robust, cost inputs were typically derived from reimbursement schedules or aggregated estimates rather than measured workflow data. Only one survey-based study reported self-reported treatment time differences. Overall, the available evidence supports system-level efficiency comparisons under specific assumptions but provides limited insight into how these findings translate into routine clinical workflows and resource demands, highlighting a mismatch between available economic evidence and the informational needs of provider-level decision-making.

### Regulatory transition and health system implementation

The EU phase-out of dental amalgam represents a regulatory intervention affecting routine clinical workflows across multiple health systems. Such transitions require economic evidence that aligns with reimbursement policy, workforce planning and service organisation.

Implementation research highlights that changes in clinical practice are shaped by organisational complexity and contextual constraints.^28 29^ While payer and societal perspectives are appropriate for macrolevel assessment,^26^ they may not capture shifts in workload or workflow complexity within dental practices. The analytic perspective chosen fundamentally determines which cost components are included and how results are interpreted.^25 30^ Misalignment between policy questions and reported perspectives may therefore limit applicability in regulatory contexts.

### Methodological heterogeneity and transferability

Substantial heterogeneity was observed across modelling approaches, perspectives, time horizons and outcome metrics, precluding quantitative synthesis. Decision-analytic models enable long-term projections but rely on structural assumptions and extrapolation from short-term clinical data.^31^ While modelling approaches inherently rely on structural assumptions, reporting on model validation (internal or external) was limited in the included studies, restricting the ability to assess structural uncertainty.

Differences in reimbursement mechanisms, insurance coverage and care pathways materially influence economic conclusions. Transferability across jurisdictions is therefore limited when cost structures and service delivery contexts differ.^26 30^

The level of costing detail also varied. Most studies relied on aggregated reimbursement data rather than explicit microcosting approaches. As highlighted in methodological literature, the distinction between gross-costing and microcosting is critical when assessing provider-level resource implications.^32^ Without transparent reporting of personnel time, equipment use or overhead allocation, operational relevance remains constrained. Reporting standards such as Consolidated Health Economic Evaluation Reporting Standards (CHEERS) 2022 may improve comparability in future research.^33^ While studies from non-EU settings were included to provide transferable insights into provider-level economic implications, differences in reimbursement systems and care delivery contexts may limit direct applicability to EU health systems.

### Implications for health systems and regulatory implementation

The amalgam phase-out requires alignment between regulatory objectives, reimbursement structures and provider incentives. Current economic evidence, largely based on modelled or reimbursement-derived inputs and supplemented by self-reported survey data, offers limited insight into measured provider-level workflow adaptation.

Where restorative alternatives involve additional procedural steps or increased clinical time, but reimbursement mechanisms do not proportionally reflect these differences, incentive misalignment may occur. The choice of analytic perspective directly determines which cost components are included and how results are interpreted.^25 30^ Such discrepancies may influence service capacity and access to care.

Short-term reintervention burden within statutory warranty periods was insufficiently itemised. In systems where early failure carries financial implications for providers, the absence of transparent retreatment data may hinder informed planning. As highlighted in methodological literature, microcosting approaches may be particularly relevant when evaluating provider-level operational effects.^32^ Our findings indicate that current studies rarely quantify practice inputs directly (eg, chair time, staff time or overhead allocation). Reporting guidance such as CHEERS 2022 and recommendations for EE highlight the importance of transparent perspective definition and detailed cost component reporting, which may improve comparability and decision relevance in future studies.^26 33^

### Strengths and limitations of this review

This review followed PRISMA-ScR guidance^20^ and applied a predefined PCC framework. Multiple databases were searched, and screening and data charting were performed independently by two reviewers.

Limitations include restriction to publications from 2021 onwards, exclusion of grey literature and absence of a formal risk-of-bias assessment consistent with scoping methodology. In particular, relevant economic evidence may be reported in grey literature sources such as NHS reports, national reimbursement analyses and EU policy assessments. These sources often provide provider-relevant cost data that are not captured in peer-reviewed publications, potentially contributing to an incomplete evidence base. Earlier EEs were not included as the focus was on contemporary restorative materials and reimbursement contexts relevant to the current EU regulatory transition. The small number of eligible studies reflects the limited economic research addressing provider-level implications in restorative dentistry. Protocol registration on OSF was retrospective, although key methodological decisions (eligibility criteria, search strategy and charting framework) were agreed before screening started.

### Implications for future research

Future research should prioritise transparent and standardised reporting of provider-level cost components. Studies should clearly define analytic perspective and time horizon, provide itemised reporting of personnel, material and overhead costs, objectively measure treatment time and workflow complexity, report reintervention within defined warranty periods and align reporting with established economic standards.^26 33^ Such improvements may enhance comparability and support evidence-informed regulatory implementation.

## Conclusions

This scoping review identified limited and methodologically heterogeneous economic evidence regarding direct posterior restorative alternatives following the EU dental amalgam phase-out. Existing studies predominantly adopt payer, societal or mixed perspectives and rely on model-based or reimbursement-based cost inputs. While such approaches inform comparative efficiency under defined assumptions, they provide limited insight into granular provider-level resource use.

Explicit microcosting of chair time, personnel allocation, overhead structures and short-term reintervention within defined warranty periods was rarely reported. Consequently, the operational and organisational implications of restorative material substitution in publicly funded health systems remain incompletely characterised.

More standardised and transparent costing could improve alignment between environmental regulation, reimbursement calibration and sustainable service delivery.

## Supplementary material

10.1136/bmjopen-2026-118949 online supplemental file 1

## Data Availability

Data sharing not applicable as no datasets generated and/or analysed for this study.

## References

[1] European Commission (2023). Mercury Regulation and dental amalgam phase-out communication. European Commission.

[2] European, Parliament Council (2024). Regulation (EU) 2024/1849 amending Regulation (EU) 2017/852 on mercury. https://eur-lex.europa.eu.

[3] United Nations Environment Programme (2019). Minamata convention on mercury.

[4] Bundesministerium der Justiz, Sozialgesetzbuch (SGB) Fünftes Buch (V) – Gesetzliche Krankenversicherung, § 136a, Germany.

[5] Opdam NJM, van de Sande FH, Bronkhorst E (2014). Longevity of posterior composite restorations: a systematic review and meta-analysis. J Dent Res.

[6] Heintze SD, Rousson V (2012). Clinical effectiveness of direct class II restorations - a meta-analysis. J Adhes Dent.

[7] Opdam NJM, Bronkhorst EM, Roeters JM (2007). Longevity of posterior amalgam and composite restorations in a general practice. J Dent Res.

[8] Beck F, Lettner S, Graf A (2015). Survival of direct resin restorations in posterior teeth. J Dent.

[9] da Veiga AMA, Cunha AC, Ferreira D (2016). Longevity of direct and indirect resin composite restorations in permanent posterior teeth: A systematic review and meta-analysis. J Dent.

[10] Worthington HV, Khangura S, Seal K (2021). Direct composite resin fillings versus amalgam fillings for permanent posterior teeth. Cochrane Database Syst Rev.

[11] Antony K, Genser D, Hiebinger C (2008). Longevity of dental amalgam compared to composite: critical review. J Dent.

[12] Hendriks FH (1985). Cost and time analysis of posterior restorations. Community Dent Oral Epidemiol.

[13] Roulet JF (1997). Benefits and disadvantages of tooth-coloured alternatives to amalgam. J Dent.

[14] Tobi H, Kreulen CM, Vondeling H (1999). Cost-effectiveness of composite resins and amalgam in the replacement of amalgam Class II restorations. Community Dent Oral Epidemiol.

[15] Schwendicke F, Basso M, Markovic D (2021). Long-term cost-effectiveness of glass hybrid versus composite in permanent molars. J Dent.

[16] Schwendicke F, Rossi JG, Krois J (2021). Cost-effectiveness of glass hybrid versus composite in a multi-country randomized trial. J Dent.

[17] Aromataris E, Stern C, Lockwood C (2022). JBI series paper 2: tailored evidence synthesis approaches are required to answer diverse questions: a pragmatic evidence synthesis toolkit from JBI. J Clin Epidemiol.

[18] Peters MDJ, Marnie C, Tricco AC (2020). Updated methodological guidance for the conduct of scoping reviews. *JBI Evid Synth*.

[19] Arksey H, O’Malley L (2005). Scoping studies: towards a methodological framework. Int J Soc Res Methodol.

[20] Tricco AC, Lillie E, Zarin W (2018). PRISMA extension for scoping reviews (PRISMA-ScR). Ann Intern Med.

[21] Bailey O, Stone SJ, Taylor G (2025). Model-Based Cost-Effectiveness of Direct Restorations: Amalgam Dominates. Community Dent Oral Epidemiol.

[22] Bailey O, Vernazza CR, Stone S (2022). Amalgam Phase-Down Part 1: UK-Based Posterior Restorative Material and Technique Use. JDR Clin Trans Res.

[23] Han A, Park EG, Yoon JH (2024). Cost-effectiveness of expanding national health insurance coverage for composite resin restorations in cancer patients in South Korea. J Dent.

[24] Usenik V, Kontić D, Kocman D (2025). Practices and attitudes of dentists regarding the use and phase-out of dental amalgam fillings: compliance with EU regulations and the Minamata Convention. *ZdravVestn*.

[25] Drummond MF, Sculpher MJ, Claxton K (2015). Methods for the Economic Evaluation of Health Care Programmes.

[26] Sanders GD, Neumann PJ, Basu A (2016). Recommendations for Conduct, Methodological Practices, and Reporting of Cost-effectiveness Analyses. JAMA.

[27] Drummond M, Barbieri M, Cook J (2009). Transferability of economic evaluations across jurisdictions. Value Health.

[28] Greenhalgh T, Robert G, Macfarlane F (2004). Diffusion of innovations in service organizations. Milbank Q.

[29] Braithwaite J, Churruca K, Ellis LA (2018). Complexity science in healthcare—aspirations, approaches, applications and accomplishments. BMJ.

[30] Walker DG, Hutubessy R, Beutels P (2010). WHO guide for standardisation of economic evaluations. Cost Eff Resour Alloc.

[31] Briggs A, Sculpher M, Claxton K (2006). Decision modelling for health economic evaluation.

[32] Tan SS, Rutten FFH, van Ineveld BM (2009). Comparing methodologies for the cost estimation of hospital services. Eur J Health Econ.

[33] Husereau D, Drummond M, Augustovski F (2022). Cheers 2022 statement. BMJ.

