# Supplementary material for: Economic evidence on provider-level implications of direct posterior amalgam alternatives following the EU phase-out: a scoping review
Source: BMJ Open. 2026 May 19;16(5):e118949. doi: 10.1136/bmjopen-2026-118949 (PMC13202131; doi:10.1136/bmjopen-2026-118949)
Supplement: online supplemental file 1 [file bmjopen-16-5-s001.docx]

**Supplementary Table S1:** Preferred Reporting Items for Systematic reviews and Meta-Analyses extension for Scoping Reviews (PRISMA-ScR) Checklist

| **Section** | **Item** | **Prisma-ScR Checklist Item** | **Reported On Page #** |
| --- | --- | --- | --- |
| **Title** | 1 | Identify the report as a scoping review. | Title page (p.1) |
| **Abstract** | 2 | Structured summary including background, objectives, eligibility criteria, sources of evidence, charting methods, results, and conclusions. | Abstract (p.1) |
| **Introduction** | 3 | Describe rationale for the review and justify scoping approach. | Introduction (p.2–3) |
|  | 4 | Explicit statement of objectives with PCC elements. | End of Introduction (p.3) |
| **Methods** | 5 | Protocol and registration. | Section 2.9 (p.6) |
|  | 6 | Eligibility criteria with rationale. | Section 2.2 (p.3–4) |
|  | 7 | Information sources and date of last search. | Section 2.3 (p.4) |
|  | 8 | Full electronic search strategy for at least one database. | Section 2.4 (p.4); Supplementary Table S2 |
|  | 9 | Selection process (screening and eligibility). | Section 2.5 (p.4); Figure 1 |
|  | 10 | Data charting process. | Section 2.6 (p.5) |
|  | 11 | Data items extracted. | Section 2.7 (p.5) |
|  | 12 | Critical appraisal rationale and method (if applicable). | Section 2.10 (p.6) |
|  | 13 | Methods of synthesis. | Section 2.8 (p.5) |
| **Results** | 14 | Numbers screened, assessed, included; reasons for exclusion; flow diagram. | Section 3.1 (p.7); Figure 1; Supplementary Tables S3–S4 |
|  | 15 | Characteristics of included sources. | Section 3.2 (p.7); Table 1 |
|  | 16 | Critical appraisal results (if done). | Not applicable |
|  | 17 | Results of individual sources of evidence. | Sections 3.3–3.4 (p.8–9); Table 3 |
|  | 18 | Summary of charting results in relation to objectives. | N/A |
| **Discussion** | 19 | Summary of evidence linked to objectives and relevance. | Sections 4.1–4.4 (p.11–12) |
|  | 20 | Limitations of the review process. | Section 4.5 (p.12–13) |
|  | 21 | Conclusions and implications. | Section 5 (p.13) |
| **Funding** | 22 | Funding sources and role of funders. | Funding statement (p.14) |

**Supplementary Table S2:** Electronic Database Search Strategy

Search period: 1 January 2021 – 22 February 2026

Languages: English, German

| **Database** | **Final Search Strategy** |
| --- | --- |
| **PubMed** | (("Composite Resins"[Mesh] OR "Glass Ionomer Cements"[Mesh] OR "Dental Amalgam"[Mesh] OR "composite resin"[tiab] OR "glass ionomer"[tiab] OR amalgam[tiab]) AND ("Dental Restoration, Permanent"[Mesh] OR "dental restoration"[tiab] OR "direct restoration"[tiab] OR "Class II"[tiab]) AND ("Costs and Cost Analysis"[Mesh] OR "cost analysis"[tiab] OR "cost-effectiveness"[tiab] OR "economic evaluation"[tiab] OR "treatment time"[tiab] OR "practice perspective"[tiab])) AND ("2021/01/01"[Date - Publication] : "2026/02/22"[Date - Publication]) |
| **Embase** | (('composite resin'/exp OR 'glass ionomer cement'/exp OR 'dental amalgam'/exp OR 'composite resin':ti,ab OR 'glass ionomer':ti,ab OR amalgam:ti,ab) AND ('dental restoration'/exp OR 'dental restoration':ti,ab OR 'direct restoration':ti,ab OR 'class ii':ti,ab) AND ('cost'/exp OR 'cost effectiveness analysis'/exp OR 'cost analysis':ti,ab OR 'cost-effectiveness':ti,ab OR 'economic evaluation':ti,ab OR 'treatment time':ti,ab OR 'practice perspective':ti,ab)) AND [2021-2026]/py |
| **Web of Science Core Collection** | TS=(("composite resin" OR "glass ionomer" OR amalgam) AND ("dental restoration" OR "direct restoration" OR "Class II") AND ("cost analysis" OR "cost-effectiveness" OR "economic evaluation" OR "treatment time" OR "practice perspective")) AND PY=(2021-2026) |
| **CINAHL** | ((TI "composite resin" OR AB "composite resin" OR TI "glass ionomer" OR AB "glass ionomer" OR TI amalgam OR AB amalgam) AND (TI "dental restoration" OR AB "dental restoration" OR TI "direct restoration" OR AB "direct restoration" OR TI "Class II" OR AB "Class II") AND (TI "cost analysis" OR AB "cost analysis" OR TI "cost-effectiveness" OR AB "cost-effectiveness" OR TI "economic evaluation" OR AB "economic evaluation" OR TI "treatment time" OR AB "treatment time" OR TI "practice perspective" OR AB "practice perspective")) Limiters: Published Date 20210101–20260222; English OR German; Peer Reviewed |
| **LIVIVO** | (("composite resin" OR "glass ionomer" OR amalgam) AND ("dental restoration" OR "direct restoration" OR "Class II") AND ("cost analysis" OR "cost-effectiveness" OR "economic evaluation" OR "treatment time")) AND publication date:[2021 TO 2026] |

The search was conducted in PubMed, Embase, Web of Science Core Collection, CINAHL, and LIVIVO to ensure comprehensive coverage of biomedical, clinical, health services, and German-language literature relevant to restorative dentistry and provider-level economic evaluation. Databases were selected to ensure coverage of biomedical, health services, and German-language literature.

**Supplementary Table S3:** Excluded full-text articles with reason

| **First Author** | **Year** | **Journal** | **Main Reason for Exclusion** | **Specific Reason** |
| --- | --- | --- | --- | --- |
| ClinicalTrials.gov registry entry | 2023 | Trial registration | No economic outcome | RCT without cost or cost-effectiveness data |
| ClinicalTrials.gov registry entry | 2024 | Trial registration | No economic outcome | Pilot survival study, no cost analysis |
| ClinicalTrials.gov registry entry | 2025 | Trial registration | No economic outcome | 12-month RCT, clinical performance only |
| Al-Asmar | 2023 | Saudi Dental Journal | No economic outcome | Replacement reasons only, no cost data |
| Alonso | 2024 | Clinical Oral Investigations | No economic outcome | Retrospective survival study |
| Alreshaid | 2023 | Journal of Dental Education | Wrong study design | Educational trends, no patient-level outcomes |
| Alreshaid | 2021 | Journal of Dental Education | Wrong study design | Educational data only |
| Balkaya | 2023 | Journal of Esthetic and Restorative Dentistry | In vitro study | Laboratory bond strength study |
| Burke | 2023 | Primary Dental Journal | Wrong population | Anterior teeth focus |
| Cavalheiro | 2025 | International Journal of Paediatric Dentistry | Wrong population | Pediatric population |
| Elkady | 2024 | Journal of Dentistry | No economic outcome | RCT, clinical performance only |
| Gupta | 2025 | Advances in Human Biology | No economic outcome | Comparative RCT without cost data |
| Hatipoğlu | 2024 | Journal of Dentistry | Wrong study design | Practice preference survey |
| Hofsteenge | 2023 | Journal of Dentistry | No economic outcome | Retrospective survival study |
| Ibrahim | 2024 | Scientific Reports | No economic outcome | RCT without cost data |
| Karaarslan | 2021 | European Journal of Prosthodontics and Restorative Dentistry | No economic outcome | Clinical comparison only |
| Khan | 2021 | Medical Forum Monthly | No economic outcome | Cross-sectional failure study |
| Mundaragi | 2024 | Int J Clin Pediatr Dent | In vitro study | Dye penetration study |
| Osiewicz | 2022 | Dental Materials | In vitro study | Laboratory wear test |
| Pozos-Guillén | 2021 | Brazilian Oral Research | Narrative review | Guideline review |
| Pradhan | 2022 | Dental Research Journal | Narrative review | Policy commentary |
| Rodrigues | 2025 | Pesquisa Brasileira em Odontopediatria e Clínica Integrada | Wrong outcome | Psychosocial outcome only |
| Schmalz | 2024 | International Dental Journal | Narrative review | Policy statement |
| Tekce | 2026 | Dental Materials | No economic outcome | Long-term RCT without cost data |
| Ulku | 2024 | Heliyon | No economic outcome | Retrospective survival study |
| Worthington | 2021 | Cochrane Database of Systematic Reviews | Not primary study | Systematic review without primary economic data |

**Supplementary Table S4:** Reasons for Exclusion at Full-Text Screening (n = 28)

| **Category** | **Definition** | **Number of Studies (n)** |
| --- | --- | --- |
| No Economic Evaluation | Studies reporting clinical performance, survival, longevity, or failure patterns without cost data, cost-effectiveness analysis, ICERs, or other economic outcomes. | 15 |
| In Vitro / Laboratory-Based Study | Experimental laboratory investigations (e.g., microleakage, bond strength, wear resistance, fracture strength) without clinical or economic endpoints. | 3 |
| Narrative Review / Policy Paper | Reviews, commentaries, or policy statements without primary data. | 4 |
| Educational / Teaching Policy Study | Studies focusing on dental school curricula, teaching allocation, or trends in material use without patient-level outcomes. | 2 |
| Provider Preference Survey | Cross-sectional surveys evaluating practitioner preferences or repair/replacement decisions without economic evaluation. | 2 |
| Wrong Population | Studies restricted to primary teeth or anterior teeth outside the review scope. | 2 |

**Note:** Some studies could theoretically fall into more than one category (e.g., technical RCT without economic outcomes). For PRISMA reporting, each study was assigned to the primary reason for exclusion to avoid double counting.
