# Supplementary material for: Economic evidence on provider-level implications of direct posterior amalgam alternatives following the EU phase-out: a scoping review
Source: BMJ Open. 2026 May 19;16(5):e118949. doi: 10.1136/bmjopen-2026-118949 (PMC13202131; doi:10.1136/bmjopen-2026-118949)
Supplement: Supplementary file 2 [file bmjopen-2026-118949.reviewer_comments.pdf]

PEER REVIEW HISTORY

BMJ Open publishes all reviews undertaken for accepted manuscripts. Reviewers are asked to complete a checklist review form and are provided with free text boxes to elaborate on their assessment. These free text comments are reproduced below.

ARTICLE DETAILS

Title (Provisional)

Economic Evidence on Provider-Level Implications of Direct Posterior Amalgam Alternatives Following the EU Phase-Out: A Scoping Review

Authors

Oesterreicher, Johannes; Herz, Marco M

VERSION 1 - REVIEW

**Reviewer**	**1**
**Name**	**Setiawan, Arlette Suzy**
**Affiliation**	**Universitas Padjadjaran**
**Date**	**12-Mar-2026**
**COI**	**None**

This manuscript presents a scoping review of the provider-level economic implications of posterior restoration alternatives after amalgam phase-out in the European Union. This topic is highly relevant due to its relevance to environmental health policy (the Minamata Convention) and its implementation in clinical dental practice.

The manuscript is structured clearly and follows PRISMA-ScR, with transparent methods and a specific focus on the granularity of provider-level costing.

However, there are several important methodological and conceptual limitations that need to be clarified before publication, particularly regarding:

• rationale for the search timeframe

• limited number of studies (only 6)

• interpretation of the results that is still too general

• potential bias due to excluding gray literature

• several minor inconsistencies in references and tables.

Major Comments

1. Search timeframe justification needs to be strengthened

The authors limited the studies to 2021–2026 to reflect more modern restorative materials.

However, this rationale is methodologically weak, as many economic evaluation studies of dental restorations were conducted before that time period.

Suggestions:

• Add stronger justification in the Methods and Discussion

• Explain that the focus is post-amalgam regulatory transition evidence

Example sentence correction:

The restriction to studies published from 2021 onward aimed to capture economic analyses reflecting contemporary restorative materials and reimbursement contexts associated with the EU amalgam phase-out policy.

2. Grey literature exclusion potentially introduces bias

The author mentions that grey literature was not systematically searched. However, in economic evaluation and policy implementation, grey literature is often crucial (e.g., NHS reports, reimbursement analyses, policy reports).

This needs to be discussed more clearly as a source of evidence gaps.

Add to the Discussion:

• NHS reports

• EU policy economic assessments

• insurance reimbursement reports

3. The number of studies is very small (n = 6)

Only six studies met the inclusion criteria. This is important, but interpretation should be more cautious.

Currently, the sentence in the Discussion is somewhat too general. It is recommended to emphasize that:

• the evidence base is still extremely limited

• most studies are based on modeling assumptions

4. The distinction between "provider-level perspective" and "provider-level costing" needs to be clarified

The manuscript distinguishes between:

• provider-level perspective

• provider-level reporting granularity

This concept is interesting, but requires more explicit conceptual clarification in the introduction to avoid reader confusion.

For example, with a brief definition:

• provider perspective = who bears the costs

• provider costing = how costs are measured operationally

5. No critical appraisal

It is permissible to omit a risk-of-bias assessment in a scoping review, but some BMJ journals often request minimal methodological reflection.

It is recommended:

Add a short paragraph explaining the general quality of the studies found, for example:

• model-based studies

• survey bias

• limited follow-up.

Minor Comments

1. Duplicate references

References 15 and 16 are identical:

Schwendicke et al. 2021 appears twice.

This should be corrected.

2. Typographical error

On the table page:

Bailey et al., 2022

should be

Bailey et al., 2022

3. PRISMA figure

In PRISMA flow diagrams, numbers in the text and diagrams should be consistent.

Example on the results page:

413 records → 279 → 34 → 6

Ensure the numbers are consistent everywhere.

4. AI statement

The AI disclosure is good but could be shortened.

The BMJ usually recommends a simple format like:

Generative AI tools were used for language editing only. The authors reviewed and approved all content.

5. Consistency of terms

The following terms should be standardized:

• “practice-level”

• “provider-level”

• “practice/provider”

Currently, they are sometimes used interchangeably.

**Reviewer**	**2**
**Name**	**Taylor, Greig**
**Affiliation**	**Newcastle University, Centre for Oral Health Research**
**Date**	**17-Mar-2026**
**COI**	**None**

Thank you for submitting this manuscript. I enjoyed reading it, but do have some comments that I think need clarified before this is accepted, and some changes that could make it a stronger paper.

- Introduction is logical, but this is very German focussed. As this ScR is about the EU, then I do wonder if more wider EU studies and considerations should be given here

- I am not sure that your justification for the range of covered studies included is strong enough. I do think you should go further back in your searches, or at least until the EU phase out was suggested

- You have no geographical restrictions, but the study is focussed on the EU phase out. I understand this, as we can learn from other areas in the world, but how does their context fit into your narrative?

- More detail about who the reviewers were at each stage, and who was the 3rd reviewer. Were they part of the authorship?

- I commend you for registering on OSF!!

- If you aren't doing ROB, then you need to cite a reference in this section 2.10 that confirms that

- Which AI tool did you use to remove duplicates, and if so, the AI section didn't report this.

- In Table,1 , the type of EE would be good e.g., CEA, CUA

- Any abbreviations in the table would benefit from being in the legend

- When you results were sensitive to parameter uncertainty, do you mean probabilistic or deterministic?

- Table 2a is in Section 3.3.1 but includes amalgam vs composite study by Bailey et al., 2025, so this needs to be adjusted

- Table 2b is redundant and I would remove

- I think you need to be clearer about how the costs in each EE were actually obtained, they were either detailed or they weren't, so be explicit (Section 3.3.4)

- Section 3.6 is totally redundant

- In section 4.3, you mention structural uncertainty remain inherent to modelling - was this found though? Were the models internally/externally validated using the appropriate tools to do so?

VERSION 1 - AUTHOR RESPONSE

Reviewer: 1

This manuscript presents a scoping review of the provider-level economic implications of posterior restoration alternatives after amalgam phase-out in the European Union. This topic is highly relevant due to its relevance to environmental health policy (the Minamata Convention) and its implementation in clinical dental practice. The manuscript is structured clearly and follows PRISMA-ScR, with transparent methods and a specific focus on the granularity of provider-level costing.

We sincerely thank the reviewer for this positive and encouraging assessment of our manuscript. We appreciate the recognition of the relevance of the topic, particularly in the context of environmental health policy and its implications for clinical dental practice. We are also grateful for the acknowledgement of the clarity of the manuscript structure, adherence to PRISMA-ScR guidance, and the focus on provider-level costing.

However, there are several important methodological and conceptual limitations that need to be clarified before publication, particularly regarding:
• rationale for the search timeframe
• limited number of studies (only 6)
• interpretation of the results that is still too general
• potential bias due to excluding gray literature
• several minor inconsistencies in references and tables.

Major Comments

1. Search timeframe justification needs to be strengthened

The authors limited the studies to 2021–2026 to reflect more modern restorative materials. However, this rationale is methodologically weak, as many economic evaluation studies of dental restorations were conducted before that time period.

Suggestions:

• Add stronger justification in the Methods and Discussion

• Explain that the focus is post-amalgam regulatory transition evidence

Example sentence correction:

The restriction to studies published from 2021 onward aimed to capture economic analyses reflecting contemporary restorative materials and reimbursement contexts associated with the EU amalgam phase-out policy.

Thank you for this important comment. We agree that the restriction to studies published from 2021 onward requires stronger justification. We have clarified this in both the Methods and Discussion sections. The timeframe was chosen to specifically capture economic evaluations reflecting contemporary restorative materials, clinical workflows, and reimbursement contexts following the regulatory momentum of the EU amalgam phase-out. We also note that the initial regulatory approach was characterised by a gradual phase-down of amalgam use, during which the broader provider-level economic implications for routine dental practice were only partially addressed. This contextual aspect has now been more explicitly reflected in the manuscript.

2. Grey literature exclusion potentially introduces bias

The author mentions that grey literature was not systematically searched. However, in economic evaluation and policy implementation, grey literature is often crucial (e.g., NHS reports, reimbursement analyses, policy reports).

This needs to be discussed more clearly as a source of evidence gaps.

Add to the Discussion:

• NHS reports

• EU policy economic assessments

• insurance reimbursement reports

We agree that the exclusion of grey literature may introduce bias, particularly in the context of economic evaluations and policy implementation. We have expanded the Discussion to explicitly acknowledge this limitation and highlight the potential relevance of sources such as

NHS reports, EU policy assessments, and insurance reimbursement analyses, which may provide practice-relevant economic data not captured in peer-reviewed literature.

3. The number of studies is very small (n = 6)
Only six studies met the inclusion criteria. This is important, but interpretation should be more cautious.
Currently, the sentence in the Discussion is somewhat too general. It is recommended to emphasize that:
• the evidence base is still extremely limited
• most studies are based on modeling assumptions

We appreciate this comment and have revised the Discussion to more cautiously interpret the findings. We now explicitly emphasize that the evidence base is very limited (n = 6) and largely based on modelling approaches rather than empirical cost data.

4. The distinction between "provider-level perspective" and "provider-level costing" needs to be clarified
The manuscript distinguishes between:
• provider-level perspective
• provider-level reporting granularity
This concept is interesting, but requires more explicit conceptual clarification in the introduction to avoid reader confusion.
For example, with a brief definition:
• provider perspective = who bears the costs
• provider costing = how costs are measured operationally

Thank you for highlighting this important conceptual issue. We have clarified the distinction in the Introduction by explicitly defining:
provider-level perspective as the analytic viewpoint (who bears the costs), and
provider-level costing as the methodological granularity of cost measurement.

5. No critical appraisal
It is permissible to omit a risk-of-bias assessment in a scoping review, but some BMJ journals often request minimal methodological reflection.
It is recommended:

We agree and have added a short paragraph discussing the general methodological characteristics and limitations of included studies (e.g., modelling approaches, limited follow-up, potential survey bias).

Add a short paragraph explaining the general quality of the studies found, for example:

• model-based studies
• survey bias
• limited follow-up.

Minor Comments

1. Duplicate references

References 15 and 16 are identical: Schwendicke et al. 2021 appears twice. This should be corrected.

Thank you for pointing this out. We have carefully reviewed the reference list and clarified that the previously duplicated entry referred to two distinct studies by Schwendicke et al. (2021). These have now been correctly distinguished as separate references (2021a and 2021b) throughout the manuscript and reference list.

2. Typographical error

On the table page: Bailey et al., 2022 should be Bailey et al., 2022

Thank you for highlighting this typographical issue. We have reviewed and corrected the formatting of the citation for Bailey et al. (2022) in the relevant table to ensure consistency with the citation style used throughout the manuscript.

3. PRISMA figure

In PRISMA flow diagrams, numbers in the text and diagrams should be consistent.

Example on the results page: 413 records → 279 → 34 → 6 Ensure the numbers are consistent everywhere.

Thank you for this comment. We have carefully verified the consistency of the study selection numbers across the manuscript text and the PRISMA flow diagram. All reported figures (records identified, screened, assessed for eligibility, and included) have been checked and aligned to ensure full consistency throughout.

4. AI statement
The AI disclosure is good but could be shortened. The BMJ usually recommends a simple format like:
Generative AI tools were used for language editing only. The authors reviewed and approved all content.

Thank you for this suggestion. The AI disclosure statement has been revised and shortened in accordance with BMJ recommendations.

5. Consistency of terms
The following terms should be standardized:
• “practice-level”
• “provider-level”
• “practice/provider”
Currently, they are sometimes used interchangeably.

Thank you for this important observation. We have standardised terminology throughout the manuscript and now consistently use the term “provider-level” to avoid ambiguity and ensure clarity.

Reviewer: 2

1. Introduction is logical, but this is very German focussed. As this ScR is about the EU, then I do wonder if more wider EU studies and considerations should be given here

We agree and have revised the Introduction to better reflect the broader EU context, including variation in reimbursement systems and implementation strategies across member states.

2. I am not sure that your justification for the range of covered studies included is strong enough. I do think you should go further back in your searches, or at least until the EU phase out was suggested

We acknowledge this point and have strengthened the justification for focusing on post-2021 evidence as representing the most policy-relevant phase of amalgam phase-out. This has been clarified in the Methods and Discussion.

3. You have no geographical restrictions, but the study is focussed on the EU phase out. I understand this, as we can learn from other areas in the world, but how does their context fit into your narrative?

We have clarified in the Methods and Discussion that non-EU studies were included to provide potentially transferable insights into provider-level economic implications, while acknowledging differences in healthcare systems. Although the focus is on the EU, evidence from other regions may still be informative, particularly given the global nature of dental material developments. We also note that substantial variation exists already across EU health systems (e.g., in financing and reimbursement), which may influence the impact of the amalgam phase-out. Accordingly, findings from both EU and non-EU studies are considered complementary.

4. More detail about who the reviewers were at each stage, and who was the 3rd reviewer. Were they part of the authorship?

We have clarified the review process in the Methods section, including the roles of all reviewers and the involvement of the third reviewer.

5. I commend you for registering on OSF!!

We sincerely thank the reviewer for this positive comment. We appreciate the recognition of our effort to improve transparency through OSF registration.

6. If you aren't doing ROB, then you need to cite a reference in this section 2.10 that confirms that
Thank you for this relevant comment. We agree that not mentioning a formal risk-of-bias assessment should be explicitly justified. For that reason, we have added a supporting reference in Section 2.10, citing established methodological guidance for scoping reviews (e.g., Arksey and O'Malley; PRISMA-ScR), which confirms that critical appraisal is not mandatory for this review type.

7. Which AI tool did you use to remove duplicates, and if so, the AI section didn't report this.

Thank you for raising this point. Duplicate removal was performed using reference management software only and did not involve the use of AI tools. Additionally, the AI

statement has also been revised to clearly indicate that generative AI tools were used exclusively for language editing and not for data processing or study selection.

8. In Table,1, the type of EE would be good e.g., CEA, CUA

We thank the reviewer for this helpful suggestion. Table 1 now includes the type of economic evaluation for each study.

9. Any abbreviations in the table would benefit from being in the legend

We have added table legends to ensure that all abbreviations are clearly defined.

10. When you results were sensitive to parameter uncertainty, do you mean probabilistic or deterministic?

We have revised the relevant sections of the manuscript (Sections 3.3 and 3.5) to improve clarity and avoid ambiguity regarding the reporting of sensitivity analyses. Specifically, we now explicitly refer to probabilistic and deterministic approaches and clarify that, while parameter uncertainty was addressed in several model-based studies, the type of sensitivity analysis was not always explicitly specified in the original publications.

11. Table 2a is in Section 3.3.1 but includes amalgam vs composite study by Bailey et al., 2025, so this needs to be adjusted

We have revised the structure of the mentioned Section. The study by Bailey et al. (2025), which evaluates amalgam versus composite restorations, has been removed from Table 2a and is now presented in Section 3.3.3, where it is discussed within the appropriate comparator group.

12. Table 2b is redundant and I would remove

Thank you for this suggestion. We agree that Table 2b did not add additional value beyond the existing presentation. The table has therefore been removed.

13. I think you need to be clearer about how the costs in each EE were actually obtained, they were either detailed or they weren't, so be explicit (Section 3.3.4)

We have revised Section 3.3.4 to more clearly describe how cost data were obtained across the included studies. Specifically, we now distinguish between model-based evaluations relying on aggregated reimbursement data or assumed cost inputs and survey-based studies reporting self-reported treatment time. We also explicitly state that no study performed detailed bottom-up micro-costing based on direct measurement of workflow inputs.

14. Section 3.6 is totally redundant

We agree that this section did not add substantial additional information beyond what was already presented. Section 3.6 has therefore been removed.

15. In section 4.3, you mention structural uncertainty remain inherent to modelling - was this found though? Were the models internally/externally validated using the appropriate tools to do so?

Thank you for this important methodological point. We have revised Section 4.3 to clarify the role of structural uncertainty in the included studies. Specifically, we now state that while decision-analytic models inherently rely on structural assumptions, reporting on model validation (internal or external) was limited or not explicitly described, restricting the ability to fully assess structural uncertainty.
